# Babies in the Corporeal Turn: The Cognitive Embodiment of Early Motor Development and Exploration in the Brazilian Context of Early Childhood Education

**DOI:** 10.5964/ejop.12143

**Published:** 2024-05-29

**Authors:** Natália Meireles Santos da Costa, Joana de Jesus de Andrade, Aline Patrícia Campos Tolentino de Lima

**Affiliations:** 1 Department of Early Childhood Education, Oslo Metropolitan University, Oslo, Norway; 2 Department of Chemistry and Education, University of São Paulo, Ribeirão Preto, Brazil; 3 Municipal Secretary of Education, Ribeirão Preto, Brazil; Tampere University, Tampere, Finland

**Keywords:** babies, early childhood education, exploration, embodiment, cultural-historical

## Abstract

The corporeal turn in developmental psychology has rekindled interest regarding how early motor development contributes to and enhances cognitive development across the first years of life. By highlighting embodied perceptual-motor engagement with the world, embodied cognitive learning emphasizes the importance of experience and perceptual-motor mechanisms in modulating the development of person-environment systems. The field currently calls for research that combines such conceptual frameworks with the complex everyday material and sociocultural landscapes that resource infants' developmental trajectories. We, therefore, aim to connect the conceptual refinement of bodily-anchored exploration to the contextual reality of everyday settings of early childhood education (ECE)—here situated in the Brazilian context—as relevant social and cultural suppliers and modulators of the developmental trajectories of babies. Secondarily, we ponder on the premises of national pedagogical curricula and their role in mediating the quality of experiences and systems of person-environment relations more closely. Cultural-historical psychology, in dialogue with the principles of Ecological Psychology, constitutes the theoretical framework that underpins the microgenetic analyses conducted. By analyzing episodes of exploratory actions of a focal baby situated in the ECE context, we seek to apprehend motor-perceptual indicators of embodied cognitive processing by considering the modes of appropriation entailed in episodes of embodied exploration. We reflect on pedagogical implications considering official national documents of early childhood education. This work contributes by providing complementary insights into the nature of infants' everyday sociocultural embodied experiences and their development in pedagogically oriented settings.

We turn our attention to an eight-month-old baby playing on the floor of an early childhood education (ECE) center. Why is she there? What does she do in that space? Which objects does she play with? What is expected of this baby in terms of development and learning? Analyzing processes of learning and appropriation entailed by educational environments requires problematizing notions that antagonize the (seemingly) private character of individual cognitive processes to (seemingly) collective material conditions of development. The current state of institutional pedagogical services for infants goes beyond providing a place to stay while families work, as it implicates ensuring the right of the child to be in that space, to be cared for, to be educated, and to develop. The shared responsibility among educators, the school community, families, and the state converges on the need to be aware of the implications of decisions related to policy, practice, and institutional provision that impact the construction of many childhoods.

## The Distinctiveness of Early Childhood

Early childhood is characterized as a period of prolonged motor and neurological immaturity and rapid learning (Tamis-LeMonda & Rodriguez, 2008). Due to high brain plasticity, the first three years of life account for the most prominent transformations in neurological development. Within this period of prolonged immaturity, survival can only be ensured by the solidarity, care, and social complementarity of the other, which the baby activates via a potent expressive and perceptual apparatus that allows intimate connection with the other and multimodal engagement with the world (Almeida, 2014; Wallon, 1959). All of this takes place in complex cultural and social environments that are the feeding sources of the development of the so-called higher psychological functions, such as thinking, memory, and language (Vygotsky, 1995). Such functions cater to the semiotic substance of cultural practices that are propagated across generations within the human realm of the world.

The combination of the above-mentioned elements accounts for early human learning and development as a highly complex period. The condition of early immaturity is disputed as a legitimate asset in relation to psychological development (Pino, 2005). As culture influences, enhances, and integrates itself to biological development (Bussab & Ribeiro, 1998), this allows the flourishment and transformation of a vast, flexible, and sophisticated repertoire of development and learning that is unparalleled to any other period of human life.

## Contributions From Cultural-Historical Psychology to Systemic and Ecological Thinking

Across various schools of thought, the field of Psychology has long grappled with dualistic divisions of human phenomena, such as the separation of nature and culture, the individual and the environment, and the mind and the body (Dennett, 1991; Gallagher, 2013; Maturana & Varela, 2001; Vygotsky, 2018; Wallon, 1959). The advocacy for overcoming such dichotomy is emphasized throughout the works of Lev Semionovich Vygotsky. The concept of “unity” recurs in various texts (Vygotsky, 1984, 2004, 2018) as an important basis for understanding the psyche as an integrated system of functions that interrelates the body, emotions, intellect, and cognition (Sawaia & Silva, 2015). Del Río and Álvarez (2007) underline that:

Vygotsky’s work could be considered as a bridge that connects the human organism to its medium, the mind to the body. It rests on the ecological, biofunctionalist and sociogenetic theories of his age, seeking within them the means of breaching the frontiers established by Cartesian thought. (p. 282)

Vygotsky’s appeal to a multidisciplinary study of the human condition (biopsychosocial-cultural) encompasses choices of words that are used to demarcate what is not only a linguistic position but also an epistemological one (Meshcheryakov, 2010). Terms such as “systems,” “unities,” “complex functions,” “experiences,” and “environment” can be understood as resources that the author employed to concretize/condense/exemplify the materiality of overcoming dichotomies and attending to integrity in the study of human development. Seeking to overcome reductionist methods, Vygotsky paved pioneering paths for the study of human development during his time and asserted the dialectical alliance between the biological, social, cultural, and historical dimensions of development. Building up on cultural-historical premises, in this paper we also engage with contemporary frameworks dedicated to the intricate subject-environment relationship, due to their updated conceptual and empirical refinement regarding early bodily development and environmental apprehension, which we now turn to.

## The Corporeal Turn and the Role of Early Motor and Perceptual Development in the Paradigm of Embodied Cognition

More recent scholarly development has underscored the nature of one’s awareness of and relation to the environment in the new light of the so-called corporeal turn (Español, 2018). Such a paradigm poses that engagement with oneself, others, and the environment can only take place through an embodied subject, which is found nested within a particular enabling organism/environment system (Adolph & Franchak, 2017; Amorim, 2011). As scholars have embraced a systemic comprehension of human development according to ecological and bodily constraints, the unity of the person-environment has been enriched by a greater consideration of both the corporeal dimension and the role of movement in advertising the nature of the psychological engagement of infants with the world (Español, 2018; Overton, 2008). This has led the status of motor development to be renovated in the field of Psychology by adding layers of complexity to the study of motor acquisition (Thelen, 1995).

Inspired by the ecological and body/mind alliance under systemic frameworks, research employing the paradigm of embodied cognition have further examined (Berger et al., 2021; Thelen, 2000) how interdependent mechanisms across locomotor onset and multiple developmental domains (such as the cognitive, social, emotional, and neurological) are related and affect development (Campos et al., 2000). Empirical findings have suggested that the process of locomotor acquisition entails a family of experiences that demand, enable, and elevate the use of more complex psychological functions, such as gestural communication, perception of self-motion, strategies of spatial coding and search, among others (Campos et al., 2000; Mulder et al., 2017).

Such a synergy between contemporary research and further conceptual elaboration has strengthened the argument that cognition is an embodied phenomenon. Being deeply grounded on the body, the integration of motor and sensorial systems modulates the awareness, engagement and learning that one can have with the world (Adolph & Franchak, 2017; Mulder et al., 2017; Thelen, 2000). As a result, it is argued that the combination of factors that compose complex psychological processes, found both in distant abstraction and here-and-now cognition, is always intricated in a bodily matrix (Berger et al., 2021; Del Río, 2002; Español, 2018; Thelen, 2000). This means that cognition caters to a larger range of everyday experiences that require and produce cognitive processing as a process distributed across the body (Thelen, 2000) and that the role of perception and action throughout motor development are key modulators in the integration of person/environment dynamics (Gibson, 1988; Thelen, 1995).

A pertinent theoretical framework within this paradigm originates from the scholarly contributions of James Gibson and Eleanor Gibson (Gibson, 1966, 1979; Gibson, 1988). Such a framework suggests that infants are deeply attuned and pursuant to environmental information through embodied mobile exploration that nests itself intimately within the dynamics of their sensorial systems. Exploration generates perceptual information related both to their own bodies as well as the environment that is codetermined by materialized action through surveillance and detection of environmental information, which has been conceptualized as the action-perception coupling (Adolph & Franchak, 2017; Gibson, 1966, 1988).

Under such terms, the conceptual complexity of motor exploration is heightened. According to Adolph and Franchak (2017), the development of action systems related to facial action (head movement and facial expressions), manual action, posture, and locomotion are highly significant to the psychological and bodily supply that infants are equipped with to perceive, plan and control interactions with their surroundings The dynamic interplay among such factors enriches the perception of opportunities for what children come to learn and do. The perception of the relationality between the organism and the environment according to the gathering and actualization of organism-environment information relates to the concept of affordance (Gibson, 1979). Affordance refers to the recognition of body/environmental information whereby unique environmental arrangements are perceived as inviting and enabling or prohibiting and disabling to a particular combination of bodily actions that might require adjustments between the organism and the environment, which operate in mutual constitution (Gibson, 1979). In this sense, exploration is a vital process for the generation of relevant environmental information for action (Adolph & Franchak, 2017; Gibson, 1988). Exploration enables perceptual learning by activating perception-action cycles which disclose affordances and allow thereby the antecipation of requirements and assessment of outcomes of specific action.

## Rationale and Aim

Given the centrality of systems of relations between the individual and the environment, advancing the study of processes of exploration and embodied manifestations of cognitive processing requires social, cultural, and temporal delimitation of the everyday contexts in which early development is rooted. In the everyday of ECE, objects are introduced, directly or indirectly to children with pedagogical purpose. Learning about and with the materiality of objects and physical furnishings implies apprehending embodied ways of acting upon them, the cultural meanings attributed by the cultural group, as well as the potential for action constructed in the intricate relationship between the subject and the object. Thus, there is the need to address the cultural inscriptions of ecological humanity embedded in the physical and semiotic mediums of pedagogical materiality (Costall, 1995; Pedersen & Bang, 2016; Tomasello, 1999). How do they both invite and are met with children’s attention and action? Hence, the social genesis of development, the symbolic materiality of technical and semiotic tools, and the inseparability between the subject and the environment serve as paradigms for understanding learning through mastering one's own conduct as a form of subjective constitution (Vygotsky, 2018).

Therefore, by combining and applying both frameworks of cultural-historical psychology and embodied cognition, this paper aims to investigate how processes of exploration entail indicators of motor-perceptual development and how such process integrates itself into how the baby experiences their bodily engagement with the contextual materiality of ECE. We recognize children's movement within their environment as the unit of experience of both lived and produced relations with others and objects.

The research reported in this paper is situated in the Brazilian context. We start by briefly presenting current Brazilian educational policies (National Curricular Guidelines for Early Childhood Education—DCNEI and the Common National Curricular Base for Early Childhood Education—BNCC-EI). We then proceed to microgenetic analysis of processual change of the motor development and exploration of a baby according to her ways of apprehending and relating to the materiality of the ECE environment. We finalize by discussing the role of the pedagogical orientation of conditions for the care and education within ECE across the first years of life (Amorim & Rossetti-Ferreira, 1999; Gobbato & Barbosa, 2017).

## Method

### The Brazilian Context of ECE

Acknowledged as a right of the child, an obligation of the state and an option of families, the segment of early childhood education in Brazil (Brasil, 1996, 1998) covers the age group from 0 to 5 years of age. Having interaction and play as its structuring axes, pedagogical practice promotes education and care as inseparable dimensions embedded in the everyday moments of pedagogically oriented environments.

Among changes in contemporary regulatory documents and policies of ECE that have impacted the work with the youngest children, we highlight the homologation of the “National Curricular Guidelines for Early Childhood Education” (DCNEI) (Brasil, 2010) and its incorporation into the National Common Curricular Base (BNCC) (Brasil, 2018). These curricular guidelines entail a pedagogical orientation focused not on learning outcomes but, instead, on the recognition of pertinent enabling competences associated with early development. As a proposal for curricular organization, the advancement of skills and abilities is posited as inherent rights within the realms of learning and development (Barbosa & Fernandes, 2020).

According to these documents (Brasil, 2010, 2018) learning rights refer to the possibilities for children and their others to live together, play, participate, explore, express, interact and learn from each other. Such rights are made possible through the organization of so-called fields of experience, namely “The I, the other and us”; “Body, gestures and movements”; “Strokes, sounds, colors, and shapes”; “Listening, speaking, thinking and imagining”; and “Spaces, times, quantities, relationships and transformations”.

The fields of experience resort to the pedagogical responsibility of educators providing and promoting enriched opportunities for babies to engage with multiple languages, ways of knowing and everyday practices. Exploration is outlined as a right of learning and development of the baby and “body, gesture and movement” composes one category of the fields of experiences that educators must attend to (Brasil, 2018). This perspective opens up interesting possibilities for the interpretation of the daycare experience, prompting us to consider what occurs in babies’ developing bodies and how this affects their engagement with objects, routines and environments within these spaces.

Therefore, having described underlying premises of the ECE context in Brazilian, we now turn to our theoretical-methodological framework.

### Theoretical-Methodological Framework

The theoretical-methodological conception of this investigation is based on historical and dialectical materialism, which also supports cultural-historical psychology. This assumption asserts that the object of study is not predetermined and defined a priori; instead, both the object and the methodology are constructed dynamically throughout the research process (Vygotsky, 1995). As Vygotsky (1995) states, the study should not “start from the object and move to its parts, but from the process to its isolated moments” (Vygotsky, 1995, p. 66). Therefore, the conversion should be from the “object to process,” which involves the study of the movement of ideas, the historical and cultural conditions, and the potentialities and limitations of subjective interpretation.

In analyzing pedagogical and social stances, the general genetic law of cultural development (Vygotsky, 1995) may or may not be realized. This law states that higher psychological functions always emerge twice: first in the collective plane of social relations (called interpsychic) and then in the individual plane (called intrapsychic) (Vygotsky, 1995, 2018). In the “unit” of an experience in educational institutions, we can identify how toys/objects, spaces, and social relationships mutually contribute to the possibility or lack thereof of cognitive development, i.e., the development of attention, perception, memory, language, logical reasoning, imagination, and affection. It is considered a premise that the environment influences a child's development in different ways at different stages of life, and at the same time, the child modifies themselves, changing their subjective constitution and, therefore, changing their relationship with the environment (Vygotsky, 2018).

Hence, the research methodology outlined here considers the context formed by policies that ensure the possibility of pedagogical practices. The choice of this methodological approach aligns with what Álvarez (1994) previously mentioned when stating that historically, research has focused on microanalyses and dyadic interactions while neglecting broader aspects that constitute the entire relationship. As a result, “The relevance both of the global systems and the historical changes in these systems for the study of psychological processes have been neglected, and consequently, a methodology accounting for this relevance has been ignored” (Álvarez, 1994, p. 29). Therefore, according to Del Río and colleagues (Del Río & Álvarez, 2007; Del Río et al., 2022), the analytical proposal considers both micro-events (focused on meaningful gestures in interaction) and macro-events (focused on objects and social practices).

### Empirical Material and Context

We present three episodes of exploration involving a focal case study of an initially 8-month-old baby whom we refer to as Sofia. The episodes are part of a doctoral research conducted by the first author of this paper (Costa, 2021) which resorts to episodes originated from video recordings of the project's database (Costa, 2021). The research was approved by a local Research Ethics Committee in Brazil (CAAE^1^1Certificates of Ethical Appraisal Submission
68655317.9.0000.5407) and had the parents' authorization for image display for academic purposes. The ECE center in question was located in a medium-sized city in Brazil. The temporal selection for this paper pertains to changes observed over the course of one month, capturing the transition from assisted sitting to initial autonomous crawling.

### Procedure of Analysis

The episodes of exploration were analyzed based on the cultural historical principles of microgenesis (Lavelli et al., 2005; Moreno-Núñez et al., 2017; Wertsch, 1985), which is an interpretation aimed at understanding the processes of appropriation considering the novelty, and the potential of experience and the dialectics of development (Vygotsky, 2018). Microgenesis entails a thematic selection of the phenomenon of interest and detailed monitoring of the minutiae of the actions of the focal subjects across the temporal course of transformation and development of the events (Góes, 2000; Rossetti-Ferreira et al., 2008). It recomposes a micro-story of the developmental trajectory by pondering the conditions in which the transformations take place. Focus is given to the interdependence and synergy among contextual conditions, interactions, modes of expression, and different actions taking place across the connection of different temporal events as something that relates to wider spheres of semiotic, cultural, and historical dimensions (Góes, 2000; Rossetti-Ferreira et al., 2008). Taking the individual and the environment as an inseparable unity, the experience apprehended in microgenetic analysis integrates action-cognition-emotion processes and manifests the refraction of the person-environment unity through the particularities of the situation being experienced (Del Río et al., 2022; Vygotsky, 1995, 2018). In terms of refining this interpretation, the challenge of identifying how this process of understanding or learning occurs in infants still requires the study of the biopsychosocial mechanisms involved.

The thematic selection of episodes (Rossetti-Ferreira et al., 2008) has employed the concepts of exploration (Gibson, 1988) and affordances (Gibson, 1979) viewed in situations of potential motor challenges for the baby. Exploration was defined as the motor-perceptual investment, particularly made visible through the use of visual and haptic strategies (Adolph et al., 2019), engendered in situations in which the baby displayed a differential investment towards an apparent challenge or focus of interest. Affordances were hinted in the analytical work by apparently prominent perception-action couplings that entailed interrelated adjustment of the action of the baby in face of the motor challenge. The orchestration of both exploration and affordances are here assumed as indicators of manifestations of embodied cognition (Thelen, 2000). Such concepts help us elucidate how the baby perceives the environment (sounds, textures, weights, contours, etc.) while simultaneously creating meanings about themselves within that environment. Hence, the analysis also draws attention to the inevitably implicated process of subjective constitution.

We shall now turn to the presentation of the empirical material. The three microgenetic episodes are presented in chronological order and articulated in discussion with pertaining issues of the frameworks of cultural historical psychology and embodied cognition. Following the episodes, considerations are made regarding implications for pedagogical practice.

## Findings and Discussion

**Figure 1:**
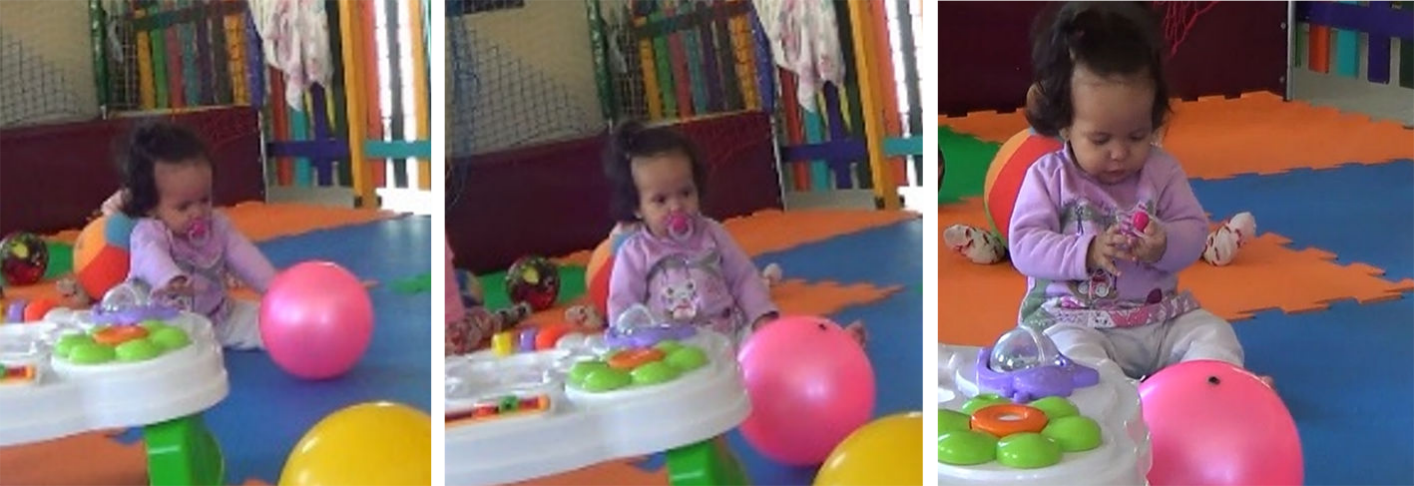
Illustration of the Episode “To Grab or Let Go of the Ball” *Note.* Source: Costa (2021).

The microgenetic process of the focal baby starts at the sitting posture. The postural base provides an axial organization for motor action (Adolph & Franchak, 2017). It is essential for aligning the head and trunk and affects how babies distribute their body weight and liberate their extremities while moving or looking around (Adolph et al., 2019). As infants learn to explore upright postures, like sitting and standing, one of their primary challenges is mastering balance over a smaller physical base (Adolph & Franchak, 2017). In one episode titled “to grab or let go of the ball” (Figure 1), we see an eight-month-old baby, Sofia, when she was becoming more experienced in sitting without back support. Such a posture was found to have been a preferential form of positioning by the different teachers in this center. The stimulation of upright posture can be attributed to cultural and socially negotiated meanings of corporeal practices proposed by traditions of education that strive to legitimize development-promoting practices in ECE. In dialectical tension, new cultural propositions confronting usual perceptual-sensory circuits, motor tonicity, and means of mobility previously experienced by the baby here produce the zone of instability and potential of transformation that constitutes the exploratory challenge (Amorim, 2011; Wallon, 1959).

As a “novice seater”, in Sofia’s experience, reaching for distal moving objects while seated was significantly challenging. She experienced restricted amplitude of back-and-forth trunk inclination, struggled with reinstating balance when changing her gravitational center. In this episode, her exploration was visible through the sustained gaze focus accompanied by slowly raising up both arms, and the stretching out both legs while inclining the trunk forward. Such exploration was deeply attuned to incidental movements of the ball. However, despite Sofia’s prolonged interest and exploratory investment, the environmental and bodily challenges were too much to overcome as the ball rolled far ahead. The whole situation seemed to enable perceptual assessment that struggles with trunk inclination and unstable balance did not afford sufficient bodily extension for reaching the now distant ball. Such a perception of (lack of) affordances seemed to lead to an abandonment of the prior exploratory investment: Sofia leaned back in straight sitting, looked around, and went on to manipulating her own pacifier, the only object at immediate reach.

The paradigm of embodied cognition proposes that across the development of new motor skills, the baby needs to constantly deal with trade-offs between attention and behavior according to tasks or perceptual information that prevails across the perception-action couplings (Adolph & Franchak, 2017; Berger et al., 2021). High investment in adjusting restrictions to balance and postural control at the cost of the pace of exploration, as described in the episode, is an example of these prepotent trade-offs (Berger et al., 2021). Such dynamics are suggested to impose an embodied cognitive load that requires the baby to actively canalize more persistent and specialized adjustments of their actions implicated with the exploratory challenge (Berger et al., 2021). However, it is not always the case that the baby is successful. In fact, errors, struggles and conflicting situations are productive virtues of the process (Adolph & Tamis-LeMonda, 2014). As the baby has the opportunity to resort to new attempts across events that reinstate themselves daily, the baby becomes more experienced and skilled by learning to regulate oneself and master the prepotent task. (Berger et al., 2021). When this happens, the baby is then able to re-allocate attentional and bodily resources to other sources of learning (Berger et al., 2021). We can observe such a description “in action” in a similar experience taking place a month later.

**Figure 2:**
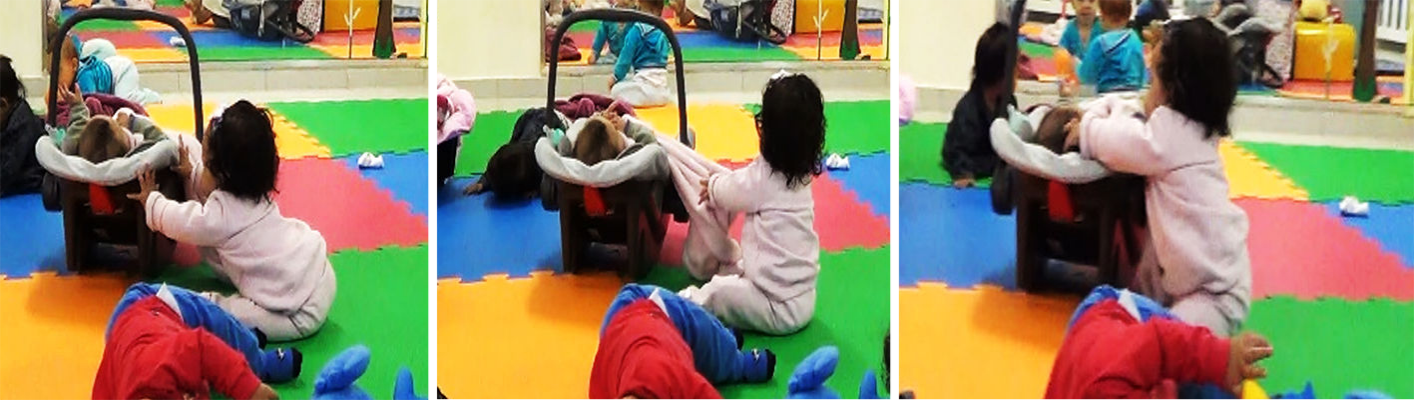
Illustration of the Episode “Pull to Move” *Note.* Source: Costa (2021).

In the episode “pull to move” (Figure 2), Sofia tackled the challenge of reaching yet another distal object, an occupied baby (car) seat. The equipment was stabilized on the ground due to the weight of another baby inside. Sofia had now developed more control, strength, and stability over the back-and-forth trunk plunging and initiated transitioning to seated crawling (Adolph, 2008). In the scene, the focal baby explored the borders of the seat via focal and distal visual alternation resourced by back-and-forth trunk inclination. When temporarily approaching the seat in deep plunging, Sofia was able to visualize a swaddling quilt placed underneath the other baby hanging over the edges of the car seat. Such an orchestration of her own bodily capacities in relation to the stable support entailed by this rather unconventional set-up afforded the quilt to function as a sort of “rope”. In the wake of this creative affordance, Sofia grabbed the blanket and, by gradual approximation, skillfully pulled herself closer to the car seat until she was close enough to caress the little head of its patient occupant.

This time, Sofia employed greater persistence towards reaching a target. She explored more creatively both her own locomotor resources as well as intermediate sources of support. Such new forms of motor action can enable features perceived both in the baby’s bodily matrix and the spatial set-up that come in intricate connection across an exploratory flow that engenders cognitive skills such as active attentional focus, means-end problem solving, spatial locations and tool use (Adolph & Franchak, 2017; Campos et al., 2000). In light of the historical construction of the microgenetic process, picking up on elements of structure, significance and dynamicity of the context helps us understand that such change is meaningful to Sofia’s development (Amorim, 2011; Del Río, 2002). The focal baby experiences a differentiated possibility to explore the space. She makes use of her own perceptual-motor capacities and embodies roles and itineraries of action that open up new possibilities of skills and affordances. Experiencing one’s own body and physical elements of the space with greater dexterity enhances pervasively the exploratory flow and transforms dialectically the relationship within the body/environment system that social and culturally feed and (re)signifies developmental trajectories.

**Figure 3:**
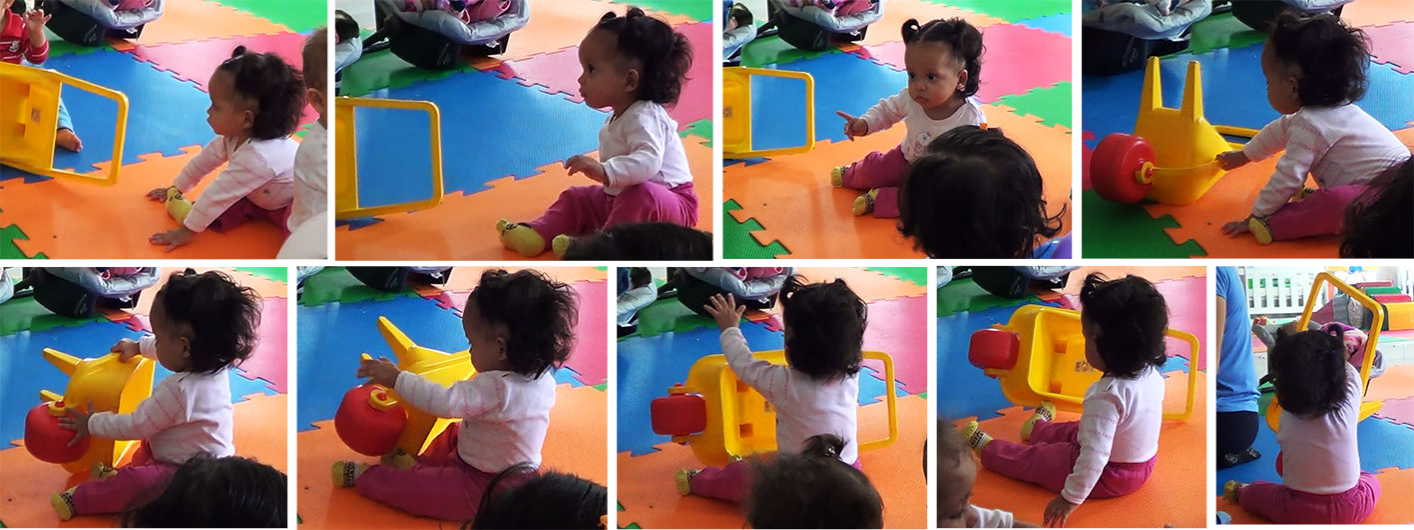
Illustration of the Episode “Take and Turn the Wagon” *Note.* Source: Costa (2021).

This renders the exploratory process a new lease of life, which we also see in the episode “take and turn the wagon”, taking place on the same day (Figure 3). After displaying conspicuous visual focus and bodily projection (Pedrosa & Carvalho, 2005) towards a wagon toy, Sofia approached it via active locomotion (seated crawling). When in possession of the toy, the baby explored multiple affordances in relation to the wagon in skillful and diversified ways. She tumbled the object over, hit the wheels, causing it to spin repeatedly, reached inside, taps the lateral border rhythmically, lifted it up with her feet, and held it up with both arms while rotating her whole body. Actions such as object rotation, high variability of hand (and feet) movement and orientation to functional features (e.g., spinning the wheel) and use of whole-body exploration are indicators of a more complex apprehension and engagement with the world. Following this prolonged bout of exploration with this single object, she put the wagon down and changed her attentional focus. Henceforth, in contrast to what we have observed initially, the exploration came full circle as Sofia was able to take initiative regarding the selection, start, development and conclusion of this exploratory episode showing greater appropriation of her actions in relation to her foci of interest.

As depicted in our findings and posited by cultural-historical psychology, the role, and the meaning of the environment vary according to the nature of the relation established between interacting factors at a given moment of their development (Pino, 2010; Vygotsky, 2018). The constitution of such relative parameters of development regards the historical process through which human semiotic heritage becomes significant to culture, and culture becomes significant to the child by means of the mediation of cultural processes (Pino, 2010; Rossetti-Ferreira et al., 2009; Vygotsky, 2018). Mediated action entails the introduction of cultural tools that provoke tensions between sociocultural provision and the concreteness of unique events that function in mutual interdependence which dialectically transform apprehension of culturally relevant bodily/environmental information that supply the formation higher psychological functions (Del Río, 2002; Valsiner, 1997; Wertsch, 1994).

Overcoming the immediacy of the senses, mediated action can perpetrate even basic motor-perceptual mechanisms that are gradually complexified. Mediational means furnish the semiotic texture of person/environment systems of development conveyed by cultural, interactive, and instrumental resources that substantiate children’s recurrent encounters with the world and the ontogeny of higher psychological functions (Del Río, 2002). As both a functional and phenomenological embodied materialization of such process (Amorim, 2011), the subtle transformation of motor action hints at trails of prior itineraries of internalization that have led to the construction of bodily mastery (re)composing what can be metaphorically described as “motor biographies” of cognitive processing. (Del Río, 2002; Wallon, 1968).

Hence, recapitulating the history of Sofia’s developmental trajectory of exploration on account of embodied cognition, the episodes depict the focal baby developing a different relation with the exploratory phenomenon itself, that is, anchored at a state of body/environment dynamic unity that affords greater mastery in taking the initiative and diversifying actions creatively. Throughout this microgenetic process, we see how exploration is processual and gradually integrated to greater body control, sharper perceptiveness, and unconventional action with objects. This dialectically engenders a transformed relation of the baby with the phenomenon of embodied exploration—here exemplified via nuances of trunk inclination, reaching and manual manipulation—as a developmental trajectory that has transitioned from hindered capacity to inaugurated possibilities of expanded motor-perceptual ways of being and acting in/with the world.

Based on the discussions presented so far, we draw to a close of this paper by suggesting pedagogical implications according to the premise of the fields of experience and the rights of learning and development.

### Final Considerations

Early childhood education has undisputed historical importance in bolstering within the societal realm the notion that children, and even babies, are historical, competent, and active subjects. Even seemingly small exploratory episodes of babies in safeguarded educational settings represent the concretization of sociohistorical transformations (Campos et al., 2001; Kuhlmann, 2015). Children are now conceived as persons deserving of rights, care, education and socialization in spaces of pedagogical quality ensured by political commitment (Brasil, 2010; Rossetti-Ferreira et al., 2009). Yet, babies are still much less visible in policy, professional development, and practice, thus urging the pedagogical work to meet the need of tailoring spaces, curricula, and educational guidance accordingly (Gobbato & Barbosa, 2017).

To effectively support babies' exploration, intentional pedagogical planning must be considerate of their diverse and complex manifestations of ways of learning and being in the world. These develop through the diversification of afforded actions, materials and physical spaces implicated in the pedagogical provision of time-spaces across the institutional every day that enrich constitutive processes of subjectivity and body awareness of the baby, both of oneself and others. The findings indicate the variability, flexibility and originality of creative solutions and exploratory investment that babies engage themselves so avidly with. According to the processes hereby discussed, voluntary motor-perceptual attention emerges as higher psychological functions through mediational means encapsulated in the dialectics of embodied relations between the person and environment (Amorim, 2011; Del Río, 2002; Vygotsky, 2018; Wertsch, 1994). Therefore, the access to more complex forms of cognitive functioning requires articulation between the values and heritage of culture (Brasil, 2018), also carved into the materiality of toys, artifacts, objects that are made available according to the regulating direction derived from official documents and policy. Pedagogical intentionality provides thereof attentional direction to the meaning and the functions of objects, things, practices, and interactive arrangements as means of enlarging cultural references of the child. It also affects children’s apprehension of their ways of knowing according to discovered interests, preferences, and curiosity about themselves and others (Oliveira et al., 2020).

Henceforth, the environment of exploration is materialized through the curative work of pedagogical intentionality coupled with how teachers, spaces, objects, and interactive practices allow (or not) certain forms of bodily engagement. Such mediational means act as transformative forces in the amplitude of experiences (Wertsch, 1994) that later converge to “... the development of thought and generalization” in the child (Vygotsky, 2018, p. 83). In this sense, we posit that the curricular matrix of ECE operates as an important consolidator of the cultural environment that pertains to the integral development of the child, even pertaining to perceptual-motor exploratory processes of embodied cognitive learning.

## Data Availability

The original dataset from the study (video recordings, interview transcripts and observation forms) has not received authorization from participants to be publicly shared. A more detailed account of the analytical script of the study is available in Portuguese in the doctoral thesis of the corresponding author at https://www.teses.usp.br/teses/disponiveis/59/59141/tde-21022022-115541/publico/Tese_Natalia_Costa_versao_corrigida.pdf
